# Discovery of the Larvae and Pupae of the Black Fly *Simulium* (*Gomphostilbia*) *khelangense* and Breeding Habitats of Potential Pest Species of the *S*. (*G*.) *chumpornense* Subgroup (Simuliidae)

**DOI:** 10.3390/insects15050346

**Published:** 2024-05-11

**Authors:** Isara Thanee, Waraporn Jumpato, Chavanut Jaroenchaiwattanachote, Bhuvadol Gomontean, Wannachai Wannasingha, San Namtaku, Peter H. Adler, Pairot Pramual

**Affiliations:** 1 Department of Biology, Faculty of Science, Mahasarakham University, Kantharawichai District, Mahasarakham 44150, Thailand; isara.th@msu.ac.th (I.T.); waraporn.a2536@gmail.com (W.J.); 64010257011@msu.ac.th (C.J.); bhuvadol.g@msu.ac.th (B.G.); wannachai.wan@msu.ac.th (W.W.); 2 Department of Science and Mathematics, Faculty of Science and Health Technology, Kalasin University, Na Mon District, Kalasin 46230, Thailand; san.na@ksu.ac.th; 3 Department of Plant and Environmental Sciences, Clemson University, Clemson, SC 29634-0310, USA; padler@clemson.edu

**Keywords:** aquatic entomology, ecology, taxonomy, insect vector

## Abstract

**Simple Summary:**

The concentration of larval black flies in well-defined aquatic habitats makes knowledge of the breeding sites critical for the efficient management of pest species. Black flies can achieve pest status either as generalists by developing in many types of streams and rivers or as specialists by developing in one or a few types of flowing water, such as large rivers. These two developmental strategies are evident in pest and vector species of the *Simulium* (*Gomphostilbia*) *varicorne* species group in Thailand. *Simulium chumpornense* is a habitat generalist, whereas *S. khelangense* is a habitat specialist, developing in the large Mekong River, where we discovered its immature stages. The first descriptions of the larva and pupa of *S. khelangense*, along with mitochondrial and nuclear genetic markers, allow accurate identification and comparisons with structurally similar species in the *S. varicorne* species group, thus aiding the ability to monitor the pest and vector status of black flies in Southeast Asia.

**Abstract:**

Two species of black flies (Simuliidae) in Thailand, *Simulium chumpornense* Takaoka and Kuvangkadilok, 2000, and *S. khelangense* Takaoka, Srisuka & Saeung, 2022, are potent vectors of avian blood protozoa of the genera *Leucocytozoon* and *Trypanosoma* and are pests of domestic avian species. Although the adults are abundant throughout Thailand, information on their breeding habitats is limited, and the immature stages of *S. khelangense* are unknown. We collected the larvae and pupae of *S. khelangense* from the Mekong River, the first-ever record of Simuliidae from this large continental river. Mitochondrial cytochrome c oxidase I and internal transcribed spacer 2 were used to associate the larvae and pupae with known adults. Both genetic markers strongly supported their identity as *S. khelangense*. The larvae and pupa of *S. khelangense* are described. The pupal gill filaments, larval abdominal protuberances, and setae distinguish this species from other members of the *S. varicorne* species group. The immature stages of *S. chumpornense* inhabit a wide variety of flowing waters, from small streams (3 m wide) to enormous continental rivers (400 m wide); thus, *S. chumpornense* is a habitat generalist. In contrast, *S. khelangense* was found only in the large Mekong River and is, therefore, a habitat specialist. Both species can exploit their principal habitats and produce abundant adult populations.

## 1. Introduction

Black flies (Diptera: Simuliidae) are significant hematophagous insects that act as pests and vectors of pathogens to humans and other animals [1]. The most significant disease associated with black flies is human onchocerciasis, caused by the filarial nematode *Onchocerca volvulus*. Black flies can also transmit viruses, bacteria, and protozoa, including those causing economically significant diseases such as leucocytozoonosis in domestic chickens [1]. Even without transmitting pathogens, black flies are significant pests of humans and other animals [1]. In some cases, biting can kill animals as a result of the toxicity of the salivary constituents, a syndrome referred to as “simuliotoxicosis” [2].

In Thailand, 145 black fly species have been recorded and are assigned to six subgenera in the genus *Simulium*: *Asiosimulium* Takaoka and Choochote; *Daviesellum* Takaoka and Adler; *Gomphostilbia* Enderlein; *Montisimulium* Rubstsov; *Nevermannia* Enderlein; and *Simulium* Latreille [3]. Females of seven species bite humans, and those of two species (*S. nigrogilvum* Summers, 1911, and *S. nodosum* Puri, 1933) are nuisance pests of humans [4]. These species, plus the *S. asakoae* Takaoka & Davies, 1995 complex, are possible vectors of filarial nematodes, including those of the genus *Onchocerca* [5,6,7,8]. Three species of the subgenus *Gomphostilbia* (*S. asakoae* complex, *S. chumpornense* Takaoka & Kuvangkadilok, 2000, and *S. khelangense* Takaoka, Srisuka & Saeung, 2022) are possible vectors of blood protozoa belonging to the genera *Leucocytozoon* and *Trypanosoma* [9,10,11]. These black flies are abundant around animal shelters and are potential pests of domestic birds [12]. 

Knowledge of the biology of insect vectors is crucial for understanding factors related to disease epidemiology and can be used for the development of effective management programs [13]. For example, the application of the highly effective larvicidal agent *Bacillus thuringiensis* var. *israelensis* (Bti) to suppress black flies requires knowledge of the breeding habitats of the target species and the physical and chemical conditions of these habitats [14,15]. Information on breeding habitats can also be used to design other management strategies, such as flow regulation [16]. 

During the past 15 years, several studies have examined habitat factors associated with species distributions of black flies in Thailand [17,18,19,20,21]. However, little is known about the breeding habitats of *S. chumpornense*. The immature stages of this species have been reported from only four stream sites, two from the southern and two from the western regions of Thailand [19], although adults are abundant throughout the country [12,22,23,24]. Another species, *S. khelangense*, is known only from the adult female [25]. Like *S. chumpornense*, this species is abundant in several areas in Thailand [11], but the breeding habitats and, therefore, the immature stages are unknown. 

We collected larvae, pupae, and data on environmental factors of the associated stream habitats. The larvae and pupae of *S. khelangense* were also collected and described for the first time. The immature stages of this species were found in the Mekong River, the first record of black flies from this enormous continental river. The characteristics of the breeding habitats of *S. chumpornense* and *S. khelangense* that are related to adult abundance are evaluated, and mitochondrial and nuclear DNA markers are assessed as aids for identification.

## 2. Materials and Methods

### 2.1. Collection and Identification

**Figure 1:**
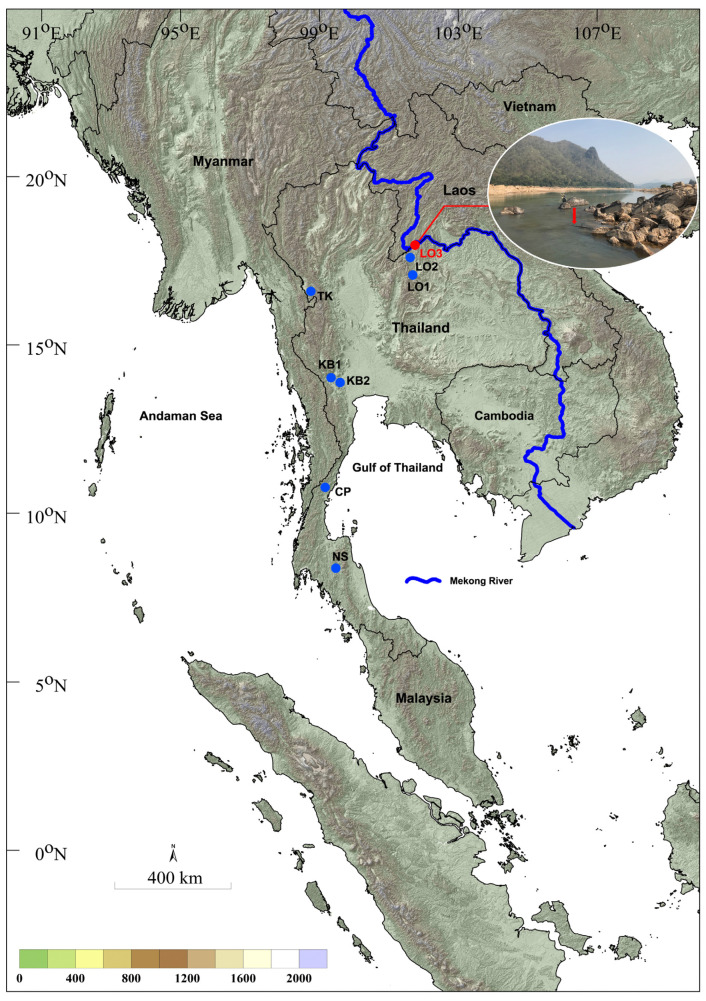
Sampling locations of larvae and pupae of *Simulium chumpornense* (blue) and S. *khelangense* (red) in Thailand. Details of sampling sites are given in Table 1. Inset shows the sampling location for larvae and pupae of *Simulium khelangense* in the Mekong River. Arrow indicates submerged wood from which larvae and pupae were collected.

Larvae and pupae were obtained from four locations (Table 1 and Figure 1). Information on stream variables reported previously [18] is included in Table 1. Larvae and pupae were collected by hand, using fine forceps, from submerged grasses and wood. Specimens were fixed in 80% (*v*/*v*) ethanol. Some pupae were reared to adults in plastic bottles. Adults were fixed in 80% ethanol for morphological study. 

The following stream variables associated with black fly distributions were measured: width, depth, velocity, conductivity, dominant streambed particle size, canopy cover, and riparian vegetation. Classification of streambed particle size, canopy cover, and riparian vegetation, plus the calculation of current velocity, followed the procedures of [26]. Larvae and pupae of the *S. varicorne* species group were identified using available keys and descriptions of black flies in Thailand [27,28,29,30,31,32,33,34,35,36,37,38,39,40]. 

### 2.2. Morphological Descriptions

The morphological characters of larvae and pupae of *S. khelangense* were examined under a stereomicroscope and compound microscope. Measurements were made using an ocular micrometer. Photographs were taken using a stereomicroscope (Zeiss Stemi 508 equipped with an Axiomcam 208 camera) and compound microscope (Zeiss PrimoStar 3 light microscope, Carl Zeiss, Germany). Descriptions of morphological characters followed the terminology of Takaoka and Suzuki [41] and Adler et al. [42]. Representative specimens were deposited in the Department of Biology, Faculty of Science, Mahasarakham University, Mahasarakham Province, Thailand. 

### 2.3. DNA Extraction, Amplification and Sequencing

DNA was extracted from the whole body of six larvae, one pupa, and one reared adult female of *S. khelangense* using the GF-1 Nucleic Acid Extraction Kit (Vivantis Technologies Sdn. Bhn, Malaysia). We also molecularly examined the closely related species *S. chumpornense* (four larvae) from a nearby location (14 km). A fragment of approximately 650 bp of the mitochondrial cytochrome c oxidase barcoding region was amplified using the primers LCO1490 (5’-GGTCAACAAATCATAAAGATATTGG-3’) and HCO2198 (5’-TAAACTTCAGGGTGACCAAAAAATCA-3’) [43]. The PCR reaction conditions followed those of [44]. In addition to the COI gene, the nuclear internal transcribed spacer 2 (ITS2) was also examined, as this genetic marker can differentiate closely related species of the *S. varicorne* species group [11]. An approximately 300-bp fragment of ITS2 was amplified using primers CP17 (5’-GCGCCGCGGTGTGAACTGCAGGACACATG-3’) and CP16 (5’-GCGGGTACCATGCTTAAATTTAGGGGGTA-3’) [45], with PCR reaction conditions as described by Thanwisai et al. [46]. The PCR products of COI and ITS2 were checked using 1% agarose gel electrophoresis staining with 1X Novel Juice Loading Dye (GenDirex^®^, Taiwan, China). Successful amplifications were purified using the PureDirex PCR CleanUp & Gel Extraction Kit (Bio-Helix, Taiwan, China). Purified PCR products were sequenced at the ATCG Company Limited (Thailand Science Park, Pathumthani, Thailand) using the same primers as for PCR.

### 2.4. DNA Sequence Analysis

A total of 12 COI (accession nos. PP564920-PP564931) and 12 ITS2 (accession nos. PP574877-PP574888) sequences (8 from *S. khelangense* and 4 from *S. chumpornense* for each gene) with a sequence length of 611 bp and 234 bp, respectively, were obtained. COI sequences (*n* = 45) of members of the *S. varicorne* species group were retrieved from GenBank and included in data analyses: *S. piroonae* Takaoka and Srisuka, 2014 (*n* = 3), *S. kuvangkadilokae* Pramual and Tangkawanit, 2008 (*n* = 16), *S. chumpornense* Takaoka and Kuvangkadilok, 2000 (*n* = 13), *S. khelangense* (*n* = 7), and *S. novemarticulatum* Takaoka and Davies, 1995 (*n* = 6). ITS2 sequences (*n* = 33) of *S. khelangense* (*n* = 18), *S. chumpornense* (*n* = 4), *S. kuvangkadilokae* (*n* = 8), and *S. novemarticulatum* (*n* = 3) were retrieved from GenBank and included for genetic distance and phylogenetic analyses. Intraspecific and interspecific genetic divergences were calculated based on a p-distance model using TaxonDNA [47]. Phylogenetic relationships were inferred using neighbor-joining (NJ), maximum likelihood (ML), and Bayesian analysis (BA) methods. NJ and ML were analyzed in MEGA X [48]. Branch support was estimated using 1000 bootstrapping replications. Bayesian analysis was performed in MrBayes ver. 3.2.7a [49] with 2,000,000 generations and a sampling frequency of 100 generations. In all phylogenetic analyses of COI and ITS2 sequences, *S. novemarticulatum,* a member of the *S. novemarticulatum* subgroup of the *S. varicorne* group [50], was treated as the outgroup. 

## 3. Results 

### 3.1. Genetic Variation, Species Differentiation, and Phylogenetic Relationships

The COI sequence divergence of larvae, pupae, and reared females of *S. khelangense* varied from 0.33% to 1.31%. Comparisons with other species of the *S. varicorne* species group revealed that these specimens were closest to *S. khelangense*, with sequence divergence ranging from 0.33% to 1.80%. This species is genetically close to *S. chumpornense*, *S. kuvangkadilokae*, and *S. piroonae*, with a minimum sequence divergence of 1.96%, 1.15%, and 2.78%, respectively. *Simulium novemarticulatum* showed a high level of genetic differentiation from the other species, with a minimum interspecific genetic divergence of 10.31% (Table 2). Genetic divergence based on ITS2 sequences showed similar patterns. The intraspecific genetic divergence of *S. khelangense* varied from 0.00% to 4.70%. Comparisons with other species revealed a considerable level of genetic differentiation, with minimum genetic divergences of 5.13% to 17.09 (Table 3).

**Figure 2:**
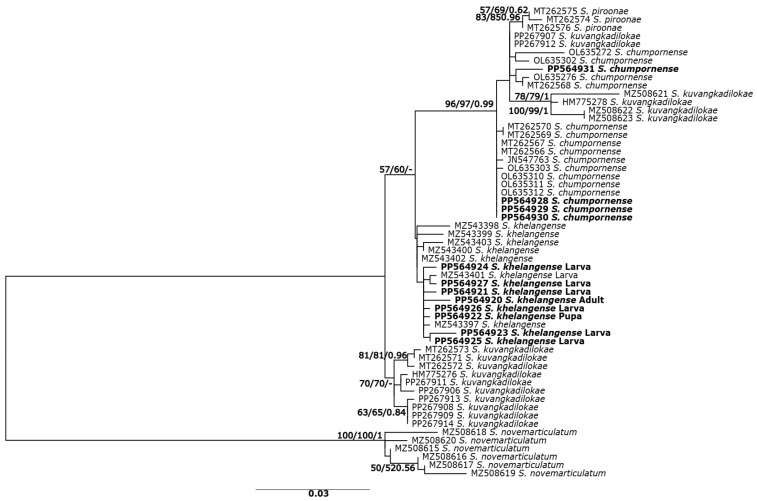
Maximum likelihood tree inferred from COI sequences of five species of the *Simulium varicorne* species group in Thailand and larvae, pupae, and reared adults of *S. khelangense.* Bootstrap values for ML and NJ analyses and posterior probability for BA trees are shown above or near branches. Bold characters indicate specimens obtained in the present study.

Phylogenetic relationships based on the COI gene sequences revealed similar tree topologies for NJ, ML, and BA methods; therefore, only the ML tree is shown (Figure 2). There were two main clades among the species. *Simulium khelangense*, *S. chumpornense*, *S. piroonae*, and *S. kuvangkadilokae* formed one clade. *Simulium khelangense* was monophyletic, although with low (<50%) support. All larvae, pupae, and reared females in our study belonged to this clade. Three specimens of *S. piroonae* were monophyletic, with >83% bootstrap support. The clade with *S. chumpornense* had strong bootstrap support (>96%) but was paraphyletic because all specimens of *S. piroonae* and 6 of 16 of *S. kuvangkadilokae* were included in this clade. Another clade comprised the remaining specimens of *S. kuvangkadilokae* with moderate bootstrap support (70%). 

**Figure 3:**
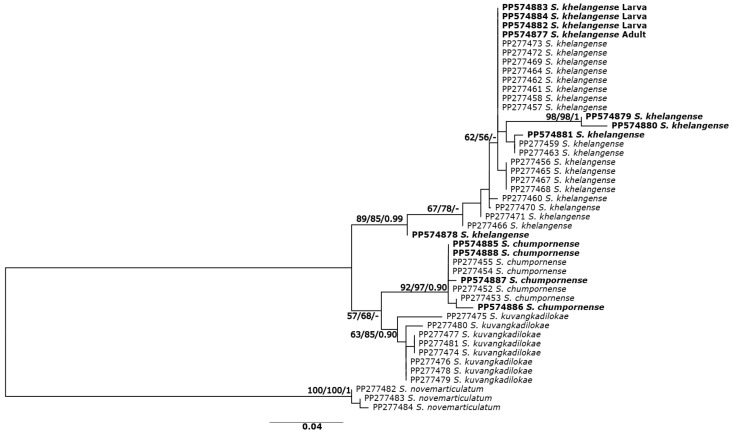
Maximum likelihood tree inferred from ITS2 sequences of five species of the *Simulium varicorne* species group in Thailand and larvae, pupae, and reared adults of *S. khelangense*. Bootstrap values for ML and NJ analyses and posterior probability for BA trees are shown above or near branches. Bold characters indicate specimens obtained in the present study.

The phylogenetic analyses based on ITS2 sequences revealed similar tree topologies for all three methods; thus, only the ML tree is presented (Figure 3). The ML tree based on ITS2 sequences revealed two clades. *Simulium khelangense* formed one clade, and *S. chumpornense* and *S. kuvangkadilokae* formed another clade. All species were monophyletic with high (>85%) support, except for *S. kuvangkadilokae*, which was low (63%) for the ML tree but high (84%) for the NJ and BA (0.9) trees. All the larvae, pupae, and reared females of *S. khelangense* clustered together.

### 3.2. Descriptions of Pupa and Larva of Simulium khelangense Takaoka, Srisuka & Saeung, 2022

Morphological comparisons of an adult female reared from a pupa agree well with the morphological characteristics of *S. khelangense* [25]. Genetic data based on COI and ITS2 sequences also indicated that the larvae and pupae collected from the Mekong River are those of *S. khelangense*. Therefore, descriptions of the mature larvae and pupa are provided here. 

**Figure 4:**
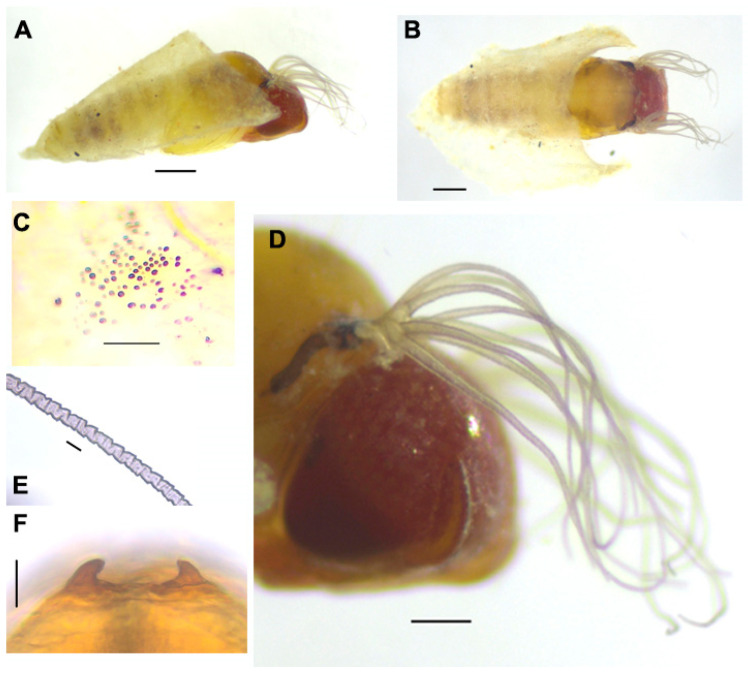
Pupa of *Simulium khelangense*. (**A**) and (**B**), pupae and cocoons ((**A**), lateral view; (**B**), dorsal view). (**C**), tubercles on the median portion of the frons. (**D**), gills (right outer side). (**E**) middle portion of the upper gill filament of the upper triplet. (**F**) terminal hooks on the dorsum of abdominal segment IX. Scale bar: 0.5 mm for (**A**) and (**B**), 0.2 mm for (**D**), 0.05 mm for (**C**) and (**E**), and 0.02 for (**F**).

**Pupa** (Figure 4). Body length (excluding gill filaments): 2.5–3.1 mm (*n* = 4) (Figure 4A,B). *Head*. Integument yellowish, moderately covered with round tubercles mostly medially and at the base of the frons (Figure 4C); antennal sheath without tubercles; frons with three pairs of unbranched long trichomes with uncoiled apices; face with a pair of unbranched long trichomes with uncoiled apices. *Thorax*. The integument is yellowish brown, moderately covered with round tubercles, with three pairs of unbranched long mediodorsal trichomes, two long unbranched anterolateral trichomes, one long unbranched posterolateral trichome, and three unbranched ventrolateral trichomes. *Gills* (Figure 4D). Each is composed of eight slender filaments arranged as 3 + (1 + 2) + 2 from dorsal to ventral; the middle triplet partially overlaps the upper triplets and lower pair; all stalks are short, although the secondary stalk of the pair of middle triplets is relatively longer than others; all filaments are light brown, subequal in thickness except for the lower filaments of the middle and lower pairs, which are slightly thicker than others; all filaments are subequal in length (ca. 1.1–1.5 mm), although the middle filament is slightly longer than others, with distinct annular ridges forming a reticulate pattern (Figure 4E), densely covered with minute tubercles. *Abdomen*. Dorsally, segment I with one simple, slender seta on each side; segment II with one simple, slender seta and five short, spinous setae submedially on each side; segments III and IV each with four hooked spines and one short spinous seta; segment V lacking spine-combs; segments VI–IX, each with a distinct spine-comb in a transverse row and with comb-like groups of minute spines on each side; segment IX with a pair of conical terminal hooks (Figure 4F). Ventrally, segment IV has one unbranched hook and a few short setae on each side; segment V has a pair of bifid hooks submedially and a few unbranched, slender short setae on each side; segments VI and VII with one inner bifid and one outer simple hook noticeably separated from each other and a few simple, slender short setae on each side; segments IV–VIII with comb-like groups of minute spines. *Cocoon*. Wall-pocket-shaped, moderately woven but more tightly woven at the anterior margin, ventrolaterally extended to varying extents, 2.5–3.2 mm long by 1.8–2.0 mm wide.

**Figure 5:**
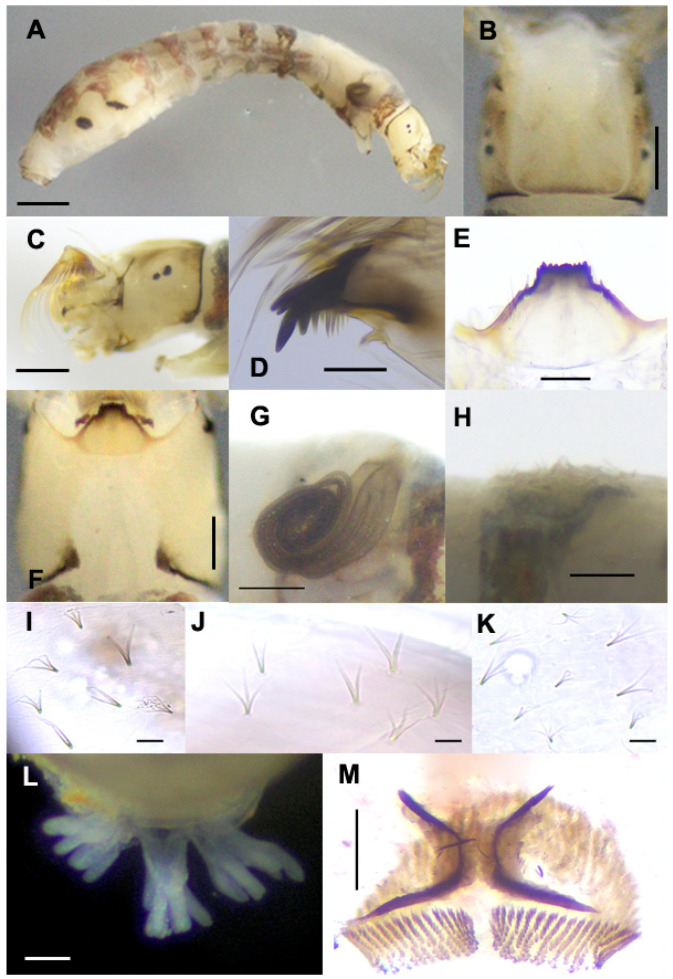
Mature larva of *Simulium khelangense*. (**A**) whole body (lateral view); (**B**,**C**) head capsule ((**B**) dorsal view; (**C**) left lateral view); (**D**) mandible; (**E**) hypostoma; (**F**) postgenal cleft; (**G**) gill histoblast; (**H**) protuberance on abdominal segment IV (lateral view); (**I**–**K**) simple, bifid, trifid, and quadrifid setae on the dorsal surface of the abdomen (dorsal view); (**L**) rectal papillae (posterodorsal view); (**M**) anal sclerite and posterior circlet. Scale bar: 0.5 mm for (**A**); 0.2 for (**B**,**C**,**F**); 0.1 for (**D**); 0.05 mm for (**E**,**G**,**H**,**L**,**M**); and 0.02 for (**I**–**K**).

**Mature Larva** (Figure 5). Body length: 4.1–4.6 mm (*n* = 10). The body is characterized by a pair of dorsolateral transparent conical protuberances on thoracic segment III and abdominal segments I–V, with those of segments III–V more prominent. Body (Figure 5A): creamy with the following markings: thoracic segment I is surrounded by a wide grayish-brown band; the anterior surface of thoracic proleg gray; thoracic segment II is whitish yellow; thoracic segment III is whitish with a dark gray spot on the ventral surface; abdominal segments I and II are each surrounded by a wide grayish brown band; abdominal segments III–VIII, each surrounded by a wide brownish band, although disconnected on abdominal segments V–VIII. The head capsule is whitish yellow except near the posterior margin of the cephalic apotome, and the upper portion above eye spots is darker (Figure 5B,C), covered with minute, simple colorless setae. The cephalic apotome (Figure 5B) is whitish-yellow with indistinct head spots, although some larvae have moderately positive patterns. The ventral surface of the head capsule is whitish yellow. Antenna unpigmented, composed of three articles and apical sensillum, slightly longer than the stem of the labral fan; proportional length of proximal, medial, and distal articles is 1.00:0.80:1.00. Labral fan with 42–44 primary rays. Mandible (Figure 5D) with three comb-teeth decreasing in length from first to third; mandibular serrations composed of two teeth, one large and one small, large tooth at a parallel angle with mandible on the apical side. Hypostoma (Figure 5E) with nine teeth, median tooth, and corner teeth prominent and subequal in length; lateral margins serrated apically; three hypostomal bristles in a row, parallel to the lateral margin on each side. The postgenal cleft is wide and deep, reaching the postgenal margin of the hypostoma (Figure 5F). Cervical sclerites are composed of two small, light brown rod-like pieces that are not fused to the occiput and are widely separated from each other. The histoblast of the pharate pupal gill has eight slender filaments (Figure 5G). Thoracic segment III and abdominal segments I–V each have a pair of conical, transparent protuberances (Figure 5H). The thoracic cuticle is bare, while the abdominal cuticle of segments I and II is sparely covered with unbranched, bifid, and trifid colorless minute setae; segments III–IV are moderately covered with minute setae; segments V–IX are densely covered with minute setae, of which unbranched setae are relatively larger with basal half or two-thirds darkened and flattened, becoming colorless and tapered apically; bifid setae are similar in length, unbranched, darker on the basal half, and colorless apically; trifid setae are shorter than unbranched and bifid setae and almost colorless excepted at base; quadrifid setae are rare, colorless, and shorter than other seta types (Figure 5I–K). Rectal papillae (Figure 5L) compound each has 7–9 finger-like secondary lobules. Anal sclerite (Figure 5M) is X-shaped, with anterior arms 0.58–0.63 times the length of the posterior arms; accessory sclerites are absent. Ventral tubercles are well developed and conical. The posterior circlet has 12–14 hooklets in 70–75 rows.

### 3.3. Diagnosis

The gill of *S. khelangense* comprises eight slender filaments in three groups (upper and middle triplets and a ventral pair) with a short common stalk (Table 4). The larva of *S. khelangense* has a pair of transparent dorsal protuberances on thoracic segment III and abdominal segments I–V and abdominal cuticle moderately covered with minute unbranched, bifid, trifid, and quadrifid setae. These morphological characteristics distinguish *S. khelangense* from all other members of the *S. varicorne* species group (Table 4).

### 3.4. Breeding Habitats of S. khelangense and S. chumpornense

The pupae and larvae of *S. khelangense* were collected from aquatic vegetation and submerged wood in the open-canopy, large (approximately 400 m wide) Mekong River (location LO3) at an elevation of 210 m above sea level. The river depth at the collection site was 0.82 m, the current velocity was 0.44 m/s, the water conductivity was 234 µS/cm^2^, and the water temperature was 23.9 °C. Larvae and pupae of *S. chumpornense* were collected from nine sampling sites; four of these were reported previously [18]. The stream size varied from 3 m to 400 m wide, with depths of 0.02 m to 0.82 m and current velocities of 0.24 m/s to 0.68 m/s. Water conductivity was generally high, with values ranging from 234 µS/cm^2^ to 495 µS/cm^2,^ and the water temperature was 22.0 °C to 32.5 °C. The dominant streambed particle size varied from sand and rubble to boulders and bedrock. Stream habitats of *S. chumpornense* were mostly without canopy cover (i.e., open), although one sampling site had a closed canopy. Riparian vegetation varied from grassland (open) to a continuous border of trees (forest) (Table 1).

## 4. Discussion

Molecular genetic analyses based on COI and ITS2 sequences confirmed that the larvae and pupae collected from the Mekong River are those of *S. khelangense*. Phylogenetic analyses based on these genetic markers further corroborated the identification of *S. khelangense*. Species of the *S. varicorne* species group are morphologically similar to adults, particularly those of the *S. chumpornense* subgroup (*S. chumpornense*, *S. kuvangkadilokae*, *S. khelangense*, *S. tomae* Takaoka, 2003, and *S. varicorne* Edwards, 1929). 

Morphological differentiation among the members of the subgroup is based mainly on the shape, number, and arrangement of the pupal gill filaments [34]. The pupal stage of *S. khelangense* can be distinguished from that of other members of the *S. varicorne* species group by the number (eight filaments), arrangement (three groups: upper and middle triplets plus a lower pair), and length (short) of the secondary stalk of the gill filaments. Among the 15 species of the *S. varicorne* species group, 10 (*S. sumbaense*, *S. chumpornense*, *S. varicorne*, *S. tomae*, *S. charlesi*, *S. novemarticulatum*, *S. huangi*, *S. shogakii*, *S. synanceium*, and *S. breviflagellum*) have eight gill filaments like *S. khelangense*. However, the gill filaments of *S. sumbaense*, *S. chumpornense*, *S. varicorne*, and *S. tomae* are arranged in two groups (six upper filaments plus a lower pair) and, therefore, differ from those of *S. khelangense*. The gill filaments of *S. charlesi*, *S. novemarticulatum*, *S. huangi*, *S. shogakii*, *S. synanceium*, and *S. breviflagellum* are arranged in three groups similar to those of *S. khelangense*. However, these species can be differentiated from *S. khelangense* by the long stalk of the ventral pair or a long secondary stalk of the upper and dorsal triplets [28,29,30,31,32,33,34,35,36,37,38,39,40]. 

The larvae of *S. khelangense* can be distinguished from those of other species in the *S. varicorne* group by the presence of a pair of transparent dorsal protuberances on thoracic segment III and abdominal segments I–V. Larvae of only five species (*S. chumpornense*, *S. piroonae*, *S. kuvangkadilokae*, *S. charlesi*, and *S. novemarticulatum*) of the members of the *S. varicorne* species group have dorsal protuberances like those of *S. khelangense*. Larvae of *S. khelangense*, which have one pair of protuberances on each of abdominal segments I–V, can be distinguished from the larvae of these species because *S. chumpornense* has two pairs [31], *S. kuvangkadilokae* has four pairs [33], and *S. piroonae* has three pairs [34] per abdominal segment. *Simulium novemarticulatum* has one pair each on abdominal segments I and II but two pairs each on abdominal segments III–V [40]. Based on the number of protuberances on the dorsal surface of the abdomen, *S. khelangense* is most similar to *S. charlesi*, with both species having a pair of dorsal protuberances on thoracic segment III and abdominal segments I–V. These species also have a similar shape to the postgenal cleft, which is wide and deep, reaching the posterior margin of the hypostoma. However, *S. khelangense* can be differentiated from *S. charlesi* by the setae on the abdominal cuticle. The abdominal cuticle of *S. khelangense* is moderately covered with minute unbranched, bifid, trifid, and quadrifid setae, whereas that of *S. charlesi* is covered with minute flat, unbranched setae [30].

We have discovered the first record of black flies inhabiting the Mekong River, the third-longest river in Asia. Three black fly species, *S. khelangense*, *S. chumpornense*, and *S. nakhonense*, were found in this river. Among these species, only *S. khelangense* and *S. chumpornense* were also found abundantly in the adult stage. The immatures of *S. chumpornense* inhabit diverse streams and rivers, ranging from small flows (3 m wide) to huge (400 m wide) continental rivers (e.g., Mekong). They can also inhabit streams with particle sizes of the streambed varying from sand to bedrock and with a canopy varying from open to completely covered. We also collected larvae of *S. chumpornense* from a highly calcareous stream (Nang Kruan waterfall, Tak Province) in the northwestern region of Thailand. Thus, this species inhabits a wide variety of streams. Therefore, as a eurytopic species occupying many habitats, *S. chumpornense* is able to produce large adult populations, one of the two major pathways to the pest status of black flies [2]. The immature stages of *S. khelangense*, on the other hand, are known only from the large Mekong River. This species, therefore, is stenotopic, able to produce large adult populations by using fewer but larger breeding habitats [2]. 

In conclusion, we have provided descriptions of the pupa and larva of *S. khelangense*. Unlike the adult female, which is difficult to distinguish from closely related species, the morphological characteristics of the pupa, particularly of the gill filaments, and of the larva, such as abdominal protuberances and setae, can be used to identify this species. The wide range of breeding habitats of *S. chumpornense* indicates that this species is a generalist. In contrast, the closely related *S. khelangense*, known only from the huge Mekong River, is a habitat specialist. Both the generalist and specialist scenarios can facilitate the production of large adult populations [2], such as those observed in several areas of Thailand. Whether these large adult populations present pest problems for avian species, such as chickens, remains to be explored.

## Figures and Tables

**Table 1:** Ecological conditions of the breeding sites of *Simulium chumpornense* and *S. khelangense* in Thailand.

Sampling Location/ Species/ Stream Parameters	Kapo, Chumporn ^1^ (CP)	Ta Pi river, Nakhon Si Thammarat ^1^ (NS)	Huai Sai, Kanchanaburi ^1^ (KB1)	Klong Phachi, Kanchanaburi ^1^ (KB2)	Nang Kruan Waterfall, Tak (TK)	Ban Kok Bok, Loei (LO1)	Na Sao, Chiang Khan, Loei (LO2)		Mekong River, Ban Pha Ban, Chiang Kan, Loei (LO3)
*S. chumpornense*	35 larvae	145 larvae	192 larvae	160 larvae	168 larvae	105 larvae	63 larvae	68 larvae	1 pupa
*S. khelangense*	-	-	-	-	-	-	-	-	139 larvae 8 pupae
Collection date	16 Aug 2001	13 Jul 2003	12 Aug 2003	12 Aug 2003	17 Dec 2015	29 Dec 2015	6 Dec 2023	17 Feb 2024	18 Feb 2024
Coordinates N, E	10.744096, 99.211731	8.526143, 99.507586	13.948365, 99.291951	13.918393, 99.382699	16.409556, 98.689278	17.392810, 101.571801	17.833940, 101.616805	17.833940, 101.616805	17.909876, 101.723808
Elevation (m)	40	40	48	48	389	274	220	220	210
Width (m)	3	15	4	3	5	4	40	40	400
Depth (m)	0.15	0.06	0.15	0.20	0.02	0.09	NA	0.20	0.82
Velocity (m/s)	NA	0.24	0.50	0.30	0.41	0.59	NA	0.68	0.44
Conductivity (µS/cm^2^)	NA	NA	495	360	512	304	398	354	234
Temperature (°C)	26.3	25.9	32.3	32.5	22.6	24.3	22.0	27.0	23.9
Streambed particle size	bedrock	sand	sand	sand	bedrock	rubble	boulders	boulders	boulders
Canopy cover	partial	open	open	open	complete	open	open	open	open
Riparian vegetation	forest	open	open	open	forest	brush	forest	forest	open

^1^ Data from [19].

**Table 2:** Intraspecific and interspecific genetic divergences (%) between species of the *Simulium varicorne* species group and the immature stages of *Simulium khelangense*, based on mitochondrial COI sequences.

Species (n)	KLIM	KL	CP	KV	PR	NO
KLIM (8)	**0.33–1.31**					
KL (7)	0.33–1.80	**0.16–1.46**				
CP (17)	1.96–3.93	1.80–3.60	**0–2.13**			
KV (16)	1.15–4.42	1.15–4.09	0.33–4.39	**0–4.58**		
PR (3)	2.78–3.76	2.62–3.44	0.65–2.13	0.33–3.93	**0.16–0.49**	
NO (6)	10.31–12.28	10.15–12.11	10.64–13.26	9.66–13.92	10.97–12.44	**0.16–2.13**

Note: KLIM, *S. khelangense* immature stage; KL, *S. khelangense* adult stage; CP, *S. chumpornense*; KV, *S. kuvangkadilokae*; PR, *S. piroonae*; NO, *S. novemarticulatum*. Bold along the diagonal indicates intraspecific genetic divergence.

**Table 3:** Intraspecific and interspecific genetic divergences (%) between species of the *Simulium varicorne* species group and the immature stages of *Simulium khelangense*, based on ITS2 sequences.

Species (n)	KLIM	KL	CP	KV	NO
KLIM (8)	**0–3.42**				
KL (18)	0–4.70	**0–2.56**			
CP (8)	5.56–9.41	5.56–8.55	**0–1.71**		
KV (16)	5.13–8.97	5.13–7.69	3.42–7.27	**0–3.42**	
NO (6)	17.09–20.51	16.67–17.95	16.67–17.52	17.09–18.38	**0.43–0.85**

Note: KLIM, *S. khelangense* immature stage; KL, *S. khelangense* adult stage; CP, *S. chumpornense*; KV, *S. kuvangkadilokae*; NO, *S. novemarticulatum*. Bold along the diagonal indicates intraspecific genetic divergence.

**Table 4:** Diagnostic morphological characters of pupae and mature larvae of all 15 species in the *Simulium varicorne* species group.

**Character/Species**	***S. breviflagellum***	***S. burtoni***	***S. charlesi***	***S. chumpornense***		***S. huangi***	***S. khelangense***	***S. kuvangkadilokae***
**Pupa**								
Number of gill filament	8	8	8	8		8	8	2 inflated + 12 threadlike
Arrangement of gill filaments	3 groups	3 groups	3 groups	2 groups		3 groups	3 groups	2 groups
Length of stalk of the ventral pair	medium-long	long	long	long		medium-long	short	short
**Larva**								
Thoracic and abdominal protuberances	NA	without	1 pair	2 pairs		without	1 pair	4 pairs
Abdominal setae	NA	simple and 2–5 branches	simple	simple and 2–4 branches		simple and 2–4 branches	simple and 2–4 branches	simple and 2–4 branches
**Character/Species**	***S. novemarticulatum***	***S. piroonae***	***S. shogakii***	***S. sumbaense***	***S. synanceium***	***S. tomae***	***S. trirugosum***	***S. varicorne***
**Pupa**								
Number of gill filament	8	6	8	8	8	8	3 inflated	8
Arrangement of gill filaments	3 groups	2 groups	3 groups	2 groups	3 group	2 groups	2 groups	2 groups
Length of stalk of the ventral pair	long	long	medium-long	long	long	long	short	long
**Larva**								
Thoracic and abdominal protuberances	1 pair	3 pairs	without	NA	NA	NA	without	NA
Abdominal setae	simple and 2–4 branches	simple and 2–4 banches	NA	NA	NA	NA	NA	NA

NA, information is not available because the larval stage is unknown, or details of abdominal setae are not given in species descriptions.

## Data Availability

The sequences have been deposited into the NCBI GenBank under the accession numbers PP564920-PP564931 and PP574877-PP574888. All other data and materials supporting this article are available from the corresponding author, P. P., upon request.
